# The relationship between triglyceride-glucose index and serum neurofilament light chain: Findings from NHANES 2013–2014

**DOI:** 10.1371/journal.pone.0321226

**Published:** 2025-04-10

**Authors:** Tong Chen, Wei Zheng, Yan Zhang, Qian Xu

**Affiliations:** 1 Department of Neurology, Xuzhou Central Hospital, Xuzhou, Jiangsu, China; 2 Department of Outpatient, Xuzhou Medical University, Xuzhou, Jiangsu, China; Tehran University of Medical Sciences, IRAN, ISLAMIC REPUBLIC OF

## Abstract

**Background:**

The Triglyceride-Glucose (TyG) index has become a reliable indicator for evaluating the level of insulin resistance, a pivotal factor in both metabolic and neurodegenerative disorders. Serum neurofilament light chain (sNfL) serves as a responsive biomarker for detecting neuroaxonal injury. Despite this, the interplay between the TyG index and sNfL levels has not been sufficiently investigated. The aim of this research is to scrutinize the correlation between TyG index and sNfL levels across a substantial, population-based cohort.

**Methods:**

Our study involved an examination of the dataset from the 2013–2014 round of the National Health and Nutrition Examination Survey (NHANES), encompassing a total of 2029 enrolled subjects. The TyG index was calculated using fasting triglycerides and glucose levels. Multivariable linear regression models were conducted to evaluate the relationship between TyG index and sNfL levels, adjusting for potential confounders such as age, sex, race, BMI, hypertension, stroke, congestive heart failure, alcohol consumption and NHHR (Non-High-Density Lipoprotein Cholesterol to High-Density Lipoprotein Cholesterol Ratio). Nonlinear associations were investigated using regression models based on restricted cubic splines (RCS).

**Results:**

Both the unadjusted and adjusted regression analyses revealed a substantial positive correlation between the TyG index and ln-sNfL levels. After accounting for all covariates, each unit increase in the TyG index was associated with a 0.15 (95% CI: 0.02–0.27, p =  0.04) increase in ln-sNfL levels. RCS analysis revealed a nonlinear relationship, with a threshold around a TyG index value of 9.63, beyond which ln-sNfL levels increased more rapidly. The association was consistent across subgroups.

**Conclusion:**

Our study links higher TyG index with increased sNfL levels, indicating insulin resistance’s role in neuroaxonal injury. The nonlinear relationship implies a heightened risk of neurodegeneration beyond a certain insulin resistance threshold. This underscores the need for early metabolic interventions to prevent neurodegenerative processes.

## Introduction

The triglyceride-glucose (TyG) index is increasingly acknowledged as a reliable proxy for assessing insulin resistance [1,2], a metabolic disorder associated with a range of illnesses, encompassing type 2 diabetes and heart and blood vessel diseases [3–5]. The role of insulin resistance in the development of neurodegenerative conditions, such as Alzheimer’s disease and Parkinson’s disease, is increasingly recognized [6–8]. Although there is a burgeoning interest in the nexus between metabolic dysregulation and neurodegeneration [9,10], the association between the TyG index and biomarkers indicative of neuroaxonal injury has not been extensively investigated in population-based research.

Serum neurofilament light chain (sNfL) is a well-established biomarker indicative of neuroaxonal injury, capturing neuronal injury across a spectrum of neurodegenerative and neuroinflammatory conditions [11,12]. Increased sNfL levels concentrations are frequently detected in individuals suffering from conditions such as Alzheimer’s disease, multiple sclerosis, and brain trauma, in addition to various other neurological afflictions [13–15]. Although sNfL is instrumental in tracking disease progression and therapeutic efficacy in these scenarios, the correlation with metabolic markers, including the TyG index, within the general population remains largely uncharted. Considering the pivotal role of insulin resistance in the genesis of both metabolic and neurodegenerative diseases, elucidating the link between the TyG index and sNfL levels may offer valuable insights into the early stages of neurodegenerative processes.

In this study, we intend to scrutinize the correlation between the TyG index and sNfL levels within a substantial, population-based dataset from the National Health and Nutrition Examination Survey (NHANES) for the 2013–2014 period. Our hypothesis posits that elevated TyG index values, signifying heightened insulin resistance, will correlate with increased sNfL levels, thereby reflecting augmented neuroaxonal damage [16,17]. Given the non-normal distribution of sNfL, a natural logarithmic transformation was implemented to normalize the data for statistical analysis [18,19]. The findings from this cross-sectional investigation may yield novel perspectives on the metabolic factors contributing to neurodegenerative pathways and facilitate the identification of preemptive biomarkers for therapeutic intervention.

## Methods

### Study population

**Fig 1:**
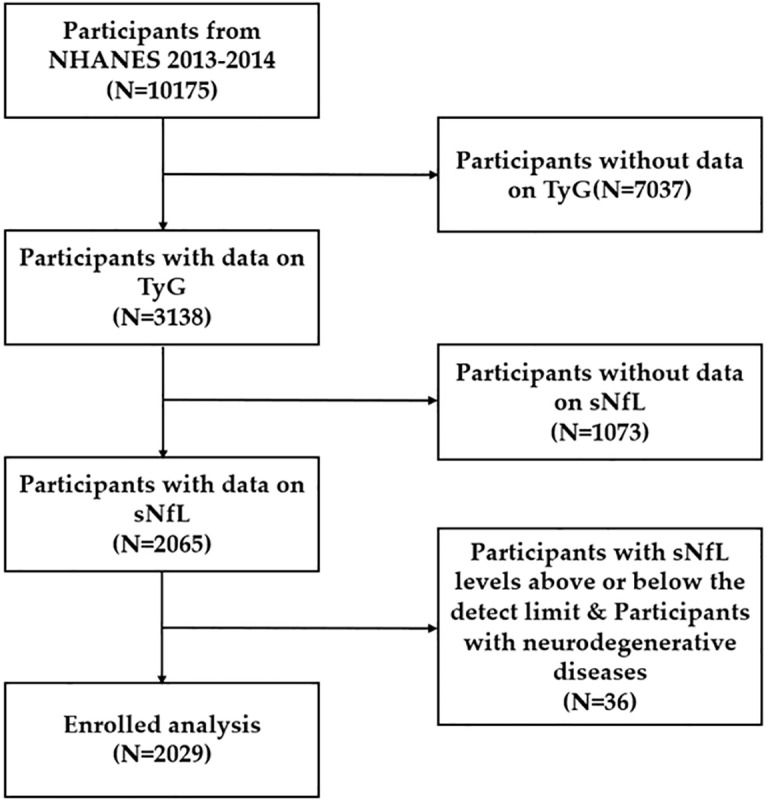
Flowchart of participant selection. NHANES, National Health and Nutrition Examination Survey guidelines.

Our research utilizes information from the 2013–2014 round of NHANES, a comprehensive, cross-sectional study aimed at evaluating the health and nutrition of the U.S. population. NHANES uses a stratified, multistage sampling approach to generate data that reflects the health status of the civilian, non-institutionalized segment of the United States. For this analysis, we included data on the TyG index and sNfL levels, as well as several covariates, including gender, age, race/ethnicity, alcohol consumption, hypertension, stroke, congestive heart failure, body mass index (BMI) and NHHR (Non-High-Density Lipoprotein Cholesterol to High-Density Lipoprotein Cholesterol Ratio). Of the initial 10,175 participants, we meticulously excluded those with missing TyG index or sNfL data, those with sNfL levels exceeding detection limits, and additionally, we systematically omitted participants diagnosed with neurodegenerative diseases to ensure the accuracy and relevance of our study’s findings. The final analysis included 2,029 participants, representing the adult U.S. population (Fig 1). The National Children’s Health and Human Development Study Ethics Review Board has authorized all protocols for the National Health and Nutrition Examination Survey (NHANES), and each survey participant has signed an informed consent form. The general public has access to comprehensive NHANES study designs and data at the official website: www.cdc.gov/nchs/nhanes/. This cross-sectional study adheres to the reporting requirements and follows the Strengthening the Reporting of Observational Studies in Epidemiology (STROBE)

### Triglyceride-glucose index calculation

The TyG index, a surrogate marker for insulin resistance, was calculated using fasting triglyceride and fasting glucose levels. The formula used to calculate the TyG index is:

$TyG=\text{ln}\left(\frac{FastingTriglycerides\left(mg/dL\right)\times FastingGlucose\left(mg/dL\right)}{2}\right)$

### Serum neurofilament light chain (sNfL) collection

The outcome variable, sNfL levels, was measured using a highly sensitive Siemens Healthineers immunoassay on the Atellica platform. The assay involves the use of acridinium ester (AE) chemiluminescence and paramagnetic particles. NfL-specific antibodies tagged with AE bind to the NfL antigen in the serum sample. Paramagnetic particles coated with capture antibodies are used to form antigen-antibody complexes, while unbound antibodies are removed to avoid interference. Chemiluminescent signals are generated by adding acid and base, with light emission proportional to the concentration of NfL in the sample. The Atellica system’s automation ensures precision and accuracy in measurements, and quality control (QC) samples at low, medium, and high concentrations were processed every 8 hours to ensure data reliability [20]. For statistical analysis, sNfL values were log-transformed (ln-sNfL) to correct for non-normal distribution.

### Covariates definition

Several covariates were included in the analysis, such as gender, age, race/ethnicity, body mass index (BMI), hypertension, stroke, congestive heart failure, alcohol consumption and NHHR (Non-High-Density Lipoprotein Cholesterol to High-Density Lipoprotein Cholesterol Ratio). Hypertension was defined based on both on-site blood pressure measurements and self-reported questionnaires. On-site blood pressure was determined by averaging three measurements. Participants were classified as hypertensive if they had a systolic blood pressure of ≥ 130 mmHg or a diastolic blood pressure of ≥ 80 mmHg. Self-reporting was ascertained through question BPQ020 (“Ever told you had high blood pressure?”). Stroke was defined using the questionnaire item MCQ160f (“Ever told you had a stroke?”). Congestive heart failure was ascertained through the questionnaire item MCQ160b (“Ever told had congestive heart failure?”). Alcohol consumption was assessed through the ALQ101 question (“Have you had at least 12 alcoholic drinks in the past year?”). NHHR was calculated as the ratio of non-high-density lipoprotein cholesterol (total cholesterol minus high-density lipoprotein cholesterol) to high-density lipoprotein cholesterol, which is used to reflect lipid levels. We categorized NHHR values into two groups based on the median: Low NHHR and High NHHR, for subsequent statistical analysis. These variables were incorporated into the analysis to control for potential confounding effects on the relationship between TyG and sNfL levels.

### Statistical methods

Data analysis was conducted using DecisionLinnc 1.0, a comprehensive software platform designed for data processing, statistical analysis, and machine learning within a graphical user interface. Categorical variables were reported as percentages, while continuous variables were assessed for adherence to a normal distribution and are reported as average values along with their standard deviations (SD). To account for the complex sampling design of NHANES, we employed a weighted nested survey design approach in our statistical analysis, incorporating survey weights and design variables. Specifically, we utilized the sample weight variable ‘WTSSNH2Y’ associated with the sNfL biomarker to conduct weighted analyses. Additionally, “SDMVPSU” was used as the sampling ID variable, and “SDMVSTRA” served as the stratification variable. This approach ensures that the estimates are reflective of the broader U.S. population, accounting for the variability and distribution of sNfL levels within the sample. The association between the TyG index and ln-sNfL levels evaluated through multiple linear regression analyses, with adjustments for possible influencing factors. Model 1 examined the crude relationship between TyG index and ln-sNfL levels. In Model 2, adjustments were made for age, sex, and race/ethnicity. In Model 3, additional adjustments were made for factors such as BMI, hypertension, stroke, congestive heart failure, and alcohol consumption to control for extra variables that could confound the results. The use of restricted cubic splines (RCS) in regression analysis allowed us to investigate possible non-linear associations between the TyG index and ln-sNfL levels, allowing for the detection of any threshold effects. Stratified analyses were performed to determine if the relationships varied among different demographic and behavioral subgroups. All analyses considered a P-value of less than 0.05 to indicate statistical significance, and 95% confidence intervals (CI) were reported for all estimates.

## Results

### Baseline characteristics of study participants

The baseline characteristics of the study participants, stratified by quartiles of the TyG index, are presented in Table 1. A total of 2,029 participants were included in the analysis. The mean age of the participants was 45.39 ± 15.10 years. Participants in the highest TyG index quartile (Q4) were older, with a mean age of 49.45 ± 13.57 years, compared to 40.90 ± 15.04 years in the lowest quartile (Q1). The mean BMI was 29.30 ± 7.31. ln-sNfL levels were higher in participants in the highest TyG index quartile (2.74 ± 0.70) compared to those in the lowest quartile (2.41 ± 0.60) (p <  0.001). In terms of sex distribution, 51.26% of the participants were female, with a higher proportion of females in the lowest TyG index quartile (61.62%) compared to the highest quartile (41.24%) (p <  0.001). In terms of lifestyle factors, 73.46% of participants reported alcohol consumption in the past year, with no significant differences across TyG index quartiles (p =  0.744). Regarding hypertension, 43.57% of the participants were hypertensive, with a significant difference across the quartiles (p <  0.001). The prevalence of hypertension was lowest in the first quartile (29.69%) and highest in the fourth quartile (63.40%). For stroke, 2.47% of the participants reported a history of stroke, with no significant difference across the quartiles (p =  0.188). Regarding congestive heart failure, 2.31% of the participants reported a history of congestive heart failure, with no significant difference across the quartiles (p =  0.102). The proportion of High NHHR increased significantly with increasing TyG index values. Specifically: Q1 (Low NHHR: 85.84%, High NHHR: 14.16%); Q4 (Low NHHR: 13.59%, High NHHR: 86.41%). A significant difference in NHHR values across quartiles was observed (p <  0.001).

**Table 1:** Mean ±  SD for continuous variables: the p-value was calculated by weighted linear regression model. % for categorical variables: the p-value was calculated by a weighted chi-square test. Q1-Q4, Equal-frequency quartile division of TyG index.

Variable	Overall	Q1(6.76–8.06)	Q2(8.06–8.49)	Q3(8.49–8.97)	Q4(8.97–12.84)	p-value
**Age (years)**	45.39 ± 15.10	40.90 ± 15.04	44.27 ± 15.05	47.56 ± 15.26	49.45 ± 13.57	<0.001
**BMI (kg/m**²)	29.30 ± 7.31	26.62 ± 6.45	28.22 ± 6.62	29.88 ± 6.98	32.84 ± 7.73	<0.001
**ln-sNfL**	2.57 ± 0.65	2.41 ± 0.60	2.53 ± 0.59	2.61 ± 0.65	2.74 ± 0.70	<0.001
**Plasma fasting glucose (mg/dl)**	105.01 ± 32.43	92.24 ± 9.26	97.90 ± 11.88	103.61 ± 19.59	128.01 ± 54.44	<0.001
**Triglycerides (mg/dl)**	120.25 ± 121.52	50.75 ± 12.75	81.98 ± 13.21	119.62 ± 21.84	238.17 ± 198.20	<0.001
**Insulin (uU/mL)**	12.77 ± 19.15	7.52 ± 6.58	10.08 ± 8.85	12.41 ± 9.66	21.67 ± 34.19	<0.001
**Sex**						<0.001
Female	51.26%	61.62%	49.78%	51.43%	41.24%	
Male	48.74%	38.38%	50.22%	48.57%	58.76%	
**Race**						<0.001
Mexican American	9.22%	5.97%	7.97%	12.17%	11.30%	
Non-Hispanic Black	11.88%	18.88%	13.69%	8.32%	5.66%	
Non-Hispanic White	65.56%	62.24%	66.81%	63.23%	70.13%	
Other Hispanic	5.63%	5.25%	5.29%	6.81%	5.27%	
Other Race	7.71%	7.66%	6.24%	9.47%	7.64%	
**Drinking behavior**						0.744
No	26.54%	26.92%	26.99%	24.38%	27.72%	
Yes	73.46%	73.08%	73.01%	75.62%	72.28%	
**Hypertension**						<0.001
No	56.43%	70.31%	61.43%	55.58%	36.60%	
Yes	43.57%	29.69%	38.57%	44.42%	63.40%	
**Stroke**						0.188
No	97.53%	97.33%	98.74%	96.47%	97.49%	
Yes	2.47%	2.67%	1.26%	3.53%	2.51%	
**Congestive heart failure**						0.102
No	97.69%	98.32%	98.92%	97.18%	96.20%	
Yes	2.31%	1.68%	1.08%	2.82%	3.80%	
**NHHR**						<0.001
Low NHHR	50.55%	85.84%	63.92%	33.64%	13.59%	
High NHHR	49.45%	14.16%	36.08%	66.36%	86.41%	

### Association between TyG index and ln-sNfL

In Model 1 (unadjusted), the TyG index was positively associated with ln-sNfL levels, with a regression coefficient (β) of 0.18 (95% CI: 0.12–0.25, p <  0.001), indicating that higher TyG index values were associated with elevated ln-sNfL levels. Once Model 2 accounted for age, gender, and racial/ethnic background, the observed relationship was modestly reduced yet continued to be statistically meaningful (β =  0.10, 95% CI: 0.04–0.15, p <  0.01). With additional considerations for BMI, hypertension, stroke, congestive heart failure, alcohol consumption and NHHR in Model 3, the significant positive relationship continued to hold (β =  0.15, 95% CI: 0.02–0.27, p =  0.04). Furthermore, trend analysis revealed a significant dose-response relationship between TyG index and ln-sNfL levels (All P for trend < 0.05) (Table 2).

**Table 2:** The association Between TyG index and sNfL levels.

Exposure	Model1 [β (95% CI)]	P-value	Model2 [β (95% CI)]	P-value	Model3 [β (95% CI)]	P-value
TyG (continuous)	0.18(0.12,0.25)	<0.001	0.10(0.04,0.15)	<0.01	0.15(0.02,0.27)	0.04
TyG (quartile)						
Q1	reference		reference		reference	
Q2	0.12(0.04,0.21)		0.05(−0.06,0.15)		0.06(−0.11,0.23)	
Q3	0.20(0.10,0.31)		0.07(–0.06,0.20)		0.08(−0.13,0.30)	
Q4	0.33 (0.21,0.45)		0.15(0.04,0.26)		0.13(−0.06,0.32)	
P for trend	<0.001		0.01		0.04	

Model 1, no covariates were adjusted. Model 2, age, sex, and race were adjusted. Model 3, age, sex, race, BMI, hypertension, stroke, congestive heart failure, alcohol consumption and NHHR were adjusted. 95% CI, 95% confidence interval; Q1-Q4, Equal-frequency quartile division of TyG index. p <  0.05 was considered statistically significant.

### Restricted cubic spline analysis

**Fig 2:**
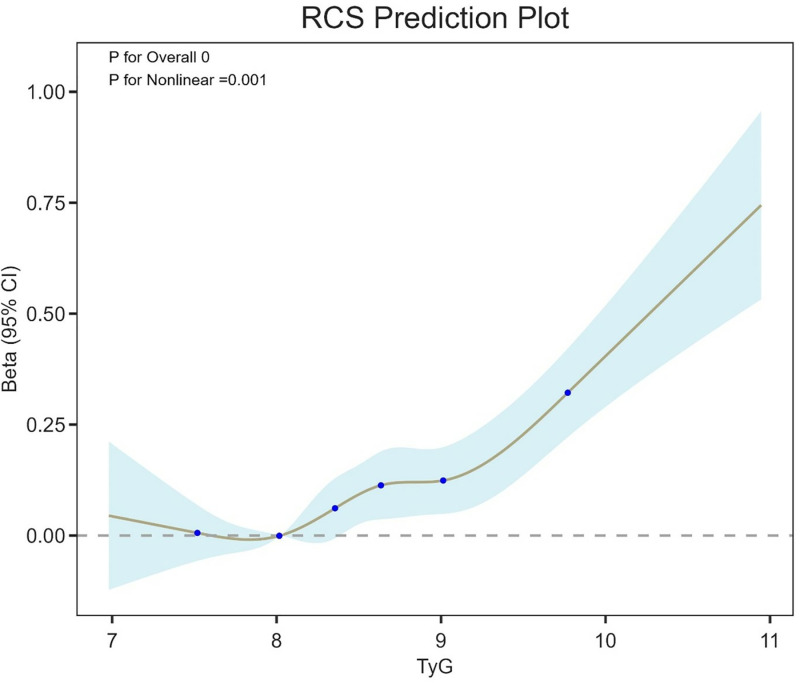
Restricted cubic spline analysis for the TyG index and sNfL levels.

To further delve deeper into the potential non-linear associations between the TyG index and ln-sNfL levels, we utilized a restricted cubic spline (RCS) analysis (Fig 2). The plot shows that the association between TyG index and ln-sNfL levels remained minimal and close to null when the TyG index was below approximately 9.63. However, beyond the threshold of 9.63, a sharp increase in ln-sNfL levels was observed with increasing TyG index values, suggesting that higher TyG index values, indicative of greater insulin resistance, were associated with significantly elevated neuroaxonal damage, as reflected by ln-sNfL levels. This result highlights a threshold effect, where neuroaxonal injury becomes more pronounced once the TyG index exceeds 9.63.

### Subgroup analysis

**Fig 3:**
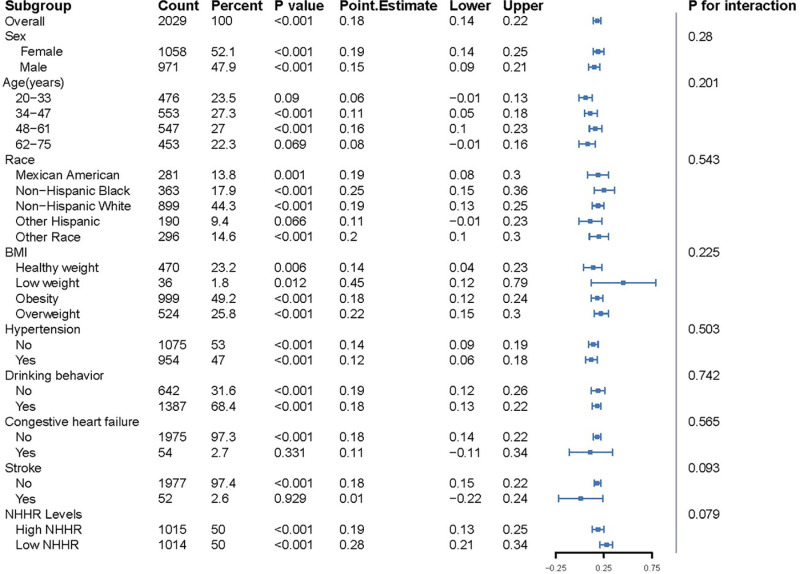
Subgroup analysis for the association between TyG index and sNfL levels.

Subgroup analyses assessed the association between the TyG index and ln-sNfL levels across various demographic, lifestyle, and chronic disease factors (Fig 3). Age subgroups were divided into four equal-width groups: 20–33 years, 34–47 years, 48–61 years, and 62–75 years. The thresholds for BMI were defined as follows: Low weight (BMI <  18.5), Healthy weight (18.5 ≤  BMI <  24.0), Overweight (24.0 ≤  BMI <  28.0), and Obesity (BMI ≥  28.0). Overall, the significant positive association between TyG index and ln-sNfL levels was consistent across multiple subgroups, and no significant interactions were found for any of the analyzed variables, indicating that the relationship between TyG index and neuroaxonal injury, as measured by ln-sNfL levels, does not differ substantially across these demographic, lifestyle, and chronic disease factors.

## Discussion

The current study evaluated the link between the TyG index, acting as a proxy for insulin resistance, and sNfL levels, a biomarker sensitive to neuroaxonal damage, based on the dataset from the 2013–2014 round of NHANES. Our findings demonstrated a strong positive correlation between the TyG index and sNfL levels, suggesting that increased insulin resistance, as signified by a higher TyG index, correlates with more extensive neuroaxonal damage. This correlation retained statistical significance after accounting for multiple potential confounding factors, such as age, sex, ethnicity, body mass index (BMI), hypertension, stroke, congestive heart failure, alcohol consumption, and NHHR. Furthermore, we discerned a nonlinear relationship between the TyG index and sNfL levels, with an inflection point at a TyG index value of approximately 9.63, above which the association between the TyG index and sNfL levels intensified.

The observed association between the TyG index and sNfL levels indicates the potential involvement of insulin resistance in the process of neuroaxonal damage, a finding consistent with prior studies that have implicated metabolic dysregulation in neurodegeneration [21–23]. One plausible mechanism underlying this association is insulin resistance’s propensity to induce chronic, low-grade inflammation [24–26]. This condition is known to activate inflammatory signaling pathways, this leads to the production and release of inflammatory signaling molecules like tumor necrosis factor-alpha (TNF-α) and interleukin-6 (IL-6) [27–29]. The presence of these inflammatory factors is associated with the development of neurodegenerative conditions, including Alzheimer’s disease and Parkinson’s disease [30–33], both characterized by elevated sNfL levels [13–15]. The persistent neuroinflammatory response elicited by insulin resistance could precipitate increased neuronal injury, as evidenced by the heightened sNfL levels in individuals presenting with higher TyG index values. An additional mechanism may involve oxidative stress and mitochondrial dysfunction. Insulin resistance has been correlated with heightened oxidative stress, which can compromise cellular components and impair mitochondrial function [34–36]. Neurons, due to their high metabolic activity, are particularly vulnerable to oxidative stress, which may precipitate neuroaxonal injury [37,38]. This mechanism could account for the positive correlation between TyG index and sNfL levels, with oxidative stress-induced neuronal damage potentially driving the observed increase in sNfL levels in individuals with elevated TyG index values. The association between the TyG index and sNfL levels could also be influenced by vascular elements. Insulin resistance is a recognized risk factor for cardiovascular disease [39–41], which shares several pathways with neurodegeneration [8,42,43]. Vascular dysfunction, including diminished cerebral blood flow and microvascular damage, may lead to neuroaxonal injury [44,45]. Elevated TyG index levels could signal subclinical vascular alterations that predispose individuals to neuronal damage, manifesting as increased sNfL levels. This highlights the possible role of vascular elements in the neurodegenerative pathways for people experiencing metabolic disorders. Lastly, blood-brain barrier (BBB) dysfunction may represent another mechanism linking insulin resistance to neuroaxonal injury. Insulin resistance has been associated with heightened BBB permeability, which typically shields the brain from detrimental circulating substances [46,47]. When the BBB’s integrity is compromised, neurotoxic agents may infiltrate the central nervous system, leading to neuronal injury [48–50]. This disruption in BBB function may partially explain the observed association between higher TyG index values and increased sNfL levels, as elevated TyG index may reflect compromised BBB function, thereby contributing to neuroaxonal damage.

Our findings have important clinical implications. The biomarker sNfL, recognized for its reliability in indicating neuronal damage, is frequently utilized to track the advancement of neurodegenerative conditions. The observed correlation between the TyG index and sNfL levels in our analysis implies that metabolic disturbances, particularly insulin resistance, may contribute to early neurodegenerative processes. The nonlinear relationship, with a threshold at a TyG index value of 9.63, suggests that the risk of neurodegeneration may accelerate once insulin resistance reaches a certain level. Regularly tracking the Triglyceride-Glucose index may assist in recognizing people who are more susceptible to neuroaxonal injury, especially in those with preexisting metabolic conditions. Moreover, strategies targeting insulin resistance reduction, including behavioral changes and medication, could potentially lessen the likelihood of neurodegenerative conditions through decreased neuroaxonal damage.

Despite the strengths of this study, such as the use of a large, nationally representative sample from NHANES and robust statistical methods, there are limitations to consider. First, The study’s cross-sectional design restricts our capacity to confirm a causal link between the TyG index and sNfL levels. Longitudinal studies are needed to confirm whether insulin resistance precedes neuroaxonal injury. Additionally, while sNfL is a sensitive marker of neuroaxonal damage, it is not specific to any particular neurodegenerative disease. Subsequent studies ought to concentrate on exploring the association between the TyG index and sNfL levels within the framework of particular neurologic disorders.

## Conclusion

Our study links higher TyG index with increased sNfL levels, indicating insulin resistance’s role in neuroaxonal injury. The nonlinear relationship implies a heightened risk of neurodegeneration beyond a certain insulin resistance threshold. This underscores the need for early metabolic interventions to prevent neurodegenerative processes.
